# Interest in fertility status assessment among young adult survivors of childhood cancer

**DOI:** 10.1002/cam4.4887

**Published:** 2022-06-01

**Authors:** Brooke Cherven, Rebecca Williamson Lewis, Megan Pruett, Lillian Meacham, James L. Klosky

**Affiliations:** ^1^ Aflac Cancer and Blood Disorders Center at Children's Healthcare of Atlanta Atlanta Georgia USA; ^2^ Department of Pediatrics Emory University School of Medicine Atlanta Georgia USA

**Keywords:** cancer survivors, fertility, reproductive health

## Abstract

**Background:**

Cancer survivors who received gonadotoxic treatment are at‐risk for future infertility and may desire a fertility status assessment (FSA), defined as semen analysis for males and consultation with a reproductive specialist for females. The purpose of this study was to describe the proportion of, and factors associated with, interest in FSA among young adult survivors of childhood cancer.

**Methods:**

This retrospective single‐institution review included patients with prior gonadotoxic treatment, aged 18–25 years and >1 year from cancer treatment completion, who received a fertility‐focused discussion during survivorship. Documentation of interest in and completion of FSA, worry about infertility, sociodemographic, and clinical characteristics were abstracted from medical records. Multivariable logistic regression was performed to calculate odds ratios (OR) and 95% confidence intervals (95%CI) for factors associated with interest in FSA.

**Results:**

Survivors (*N* = 259) were on average 19.2 ± 1.2 years at their fertility discussion; 55.6% were male and 57.9% non‐Hispanic white. Interest in FSA was reported by 50.7% of males and 46.1% of females. Factors related to interest in FSA for males and females respectively, included worry about infertility (OR 2.40, 95%CI 1.11–5.27, *p* = 0.026 and OR 4.37, 95%CI 1.71–12.43, *p* = 0.003) and ≥2 fertility discussions (OR 3.78, 95%CI 1.70–8.75, *p* = 0.001 and 2.45, 95%CI 1.08–5.67, *p* = 0.033). Among males, fertility preservation consult/procedure at diagnosis (OR 3.02, 95%CI 1.09–9.04, *p* = 0.039) and high‐risk for infertility (OR 2.47, 95%CI 1.07–5.87, *p* = 0.036) were also associated with interest in FSA.

**Conclusions:**

Cancer survivors are interested in FSA, particularly those who have had repeated fertility‐focused discussions during survivorship care and who report worry about infertility.

## Graphical abstract

For survivors of childhood cancer who are at risk for infertility, a fertility status assessment can provide clarity regarding their current fertility status and options for future biological parenthood. Cancer survivors are interested in fertility status assessment, particularly those who have had repeated fertility‐focused discussions during survivorship care and those who report worry about infertility. Despite interest in a fertility status assessment, few survivors have completed an assessment.

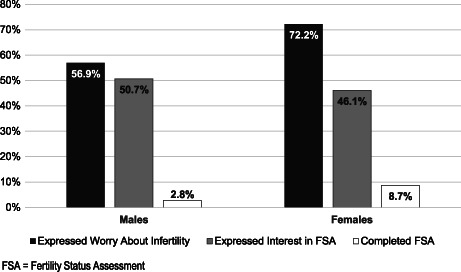

## 1 INTRODUCTION

Nearly 85% of children and adolescents diagnosed with cancer will survive,^1^ however many are exposed to gonadotoxic treatment as part of their cancer therapy and are therefore at risk for infertility.^2^ Receipt of gonadotoxic therapies (e.g., alkylating or heavy metal chemotherapy, radiation and/or surgery impacting the gonads, radiation to the hypothalamus, and/or hematopoietic stem cell transplant) is associated with oligospermia or azoospermia in males^3^, ^4^ and premature ovarian insufficiency or primary ovarian failure among females.^2^, ^5^ Infertility rates among childhood cancer survivors range from 42–66% for males and 11–25% for females.^6^

Male and female young adult survivors of childhood cancer (YASCC) can experience distress regarding potential infertility.^7^, ^8^, ^9^ Discussions about future fertility are a priority among patients and families at the time of cancer diagnosis^10^, ^11^ and during survivorship.^12^, ^13^, ^14^ Fertility‐related concerns (e.g., uncertainty regarding fertility, worry about pregnancy or future parenthood^15^, ^16^) are reported by 20–60% of cancer survivors.^7^ Fertility‐related concerns have been associated with psychological distress, depression, and anxiety,^7^ and desiring a child or another child are associated with increased distress and poorer mental health.^17^, ^18^, ^19^ Most survivors in emerging adulthood (i.e., 18–25 years of age) are unlikely to have had children and may experience fertility‐related distress.

Although infertility is a significant concern for YASCC, many are unaware of their fertility status as they have neither been formally assessed nor pursued a natural pregnancy.^20^ Among survivors at‐risk for infertility, a fertility status assessment (FSA) is frequently recommended to those who are interested, consistent with national guidelines.^21^ FSA in females usually involves the interpretation of hypothalamic‐pituitary‐ovarian hormones and an antral follicle count^22^ interpreted by a fertility specialist (e.g., reproductive endocrinologist). Fertility status for males is most accurately assessed through semen analysis.^23^

Survivors who have received gonadotoxic cancer therapy desire personalized information regarding their risk for infertility^24^ and current fertility status.^25^, ^26^ In a sample of 98 cancer survivors in early adulthood (18–21 years of age), 71% of females and 66% of males reported interest in FSA,^27^ yet studies of adult (18–55 years of age) cancer survivors demonstrate low uptake of semen analysis among males and evaluation by a fertility specialist among females.^28^, ^29^, ^30^, ^31^ In a study of 92 survivors of childhood cancer (22–44 years of age), participants reported uncertainty and concerns regarding fertility, yet almost half had not completed an FSA; younger age was associated with non‐completion of an FSA.^32^ While the provision of tailored education has proven successful in improving accurate knowledge of infertility risk,^27^ little is known regarding factors that influence survivors' interest in, and receipt of, FSA during early young adulthood.

Survivors of childhood cancer typically face a healthcare transition from pediatric to adult healthcare systems during young adulthood. It is unclear what proportion of survivors are interested in an FSA during emerging adulthood, while under the care of a pediatric cancer survivorship program. The purpose of this study was to describe the proportion of YASCC interested in and completing, FSA. We also describe clinical and sociodemographic factors associated with FSA interest (defined as a semen analysis in males, or formal consultation with a reproductive endocrinologist accompanied by hormone assessment and/or antral follicle count for females).

## 2 METHODS

This study utilized a single institution review of clinical data abstracted from electronic medical records among YASCC who received care within the childhood cancer survivor clinic. The multidisciplinary cancer survivor clinic includes oncology, endocrinology, psychology, nursing, and social work providers who see patients beginning around 2 years after completion of their cancer treatment. Survivors are followed until the age of 21 years at which time they transition to adult healthcare; patients over the age of 21 years who have never attended the survivor clinic are eligible for a one‐time visit. Eligibility criteria for this study included (1) prior cancer diagnosis; (2) ≥1 visit to the cancer survivor clinic between 1/1/2017 and 9/30/2019; (3) ≥1 year from completion of cancer treatment; (4) 18.00–25.99 years of age at the time of visit; (5) and a history of receiving gonadotoxic therapy (alkylating agents, heavy metal chemotherapy, radiation affecting the gonads [pelvis, spine, total body irradiation, cranial radiation ≥30 Gy], retroperitoneal lymph node dissection and/or hematopoietic cell transplant). Survivors were excluded if they had not received a fertility‐focused discussion during their survivorship clinical visit. Additionally, if survivors had received their initial fertility‐focused discussion <18 years of age, they were excluded due to differences in the developmental stage and decision‐making capacity for pursuing FSA. Survivors of brain tumors are followed in a separate survivorship clinic at the study institution and are not represented in this study. Ethical approval for this study was obtained from the Children's Healthcare of Atlanta institutional review board (IRB# 00000416).

Consistent with national guidelines, survivors who received gonadotoxic treatment were counseled regarding their risk for infertility with a focused discussion at age 18 years or older. Survivor team endocrinology providers led a discussion of the survivor's risk for treatment‐related infertility, an interpretation of current reproductive hormone levels, and an assessment of fertility‐related worry. The discussion also included options for FSA and family building and interested survivors were provided with a handout with contact information for reproductive clinics where they could complete an FSA; for survivors who expressed interest in an FSA, this was documented in the medical record. Receipt of FSA was documented in the clinical record based on survivor reports at subsequent clinical visits, or if the FSA report was sent to our center, and in some cases, the fertility team conducted follow‐up phone calls with survivors.

### 2.1 Variables of interest

#### 2.1.1 Sociodemographic and clinical

Sociodemographic variables were abstracted from participant medical records, including sex, race/ethnicity, insurance type, religion, and zip code. Using zip codes, participants were classified as living in rural or non‐rural areas in accordance with the Health Resources and Services Administration Federal Office of Rural Health Policy.^33^ Clinical characteristics included cancer diagnosis category (leukemia, lymphoma, solid tumor), age at diagnosis, and time from treatment completion (in years). Treatment‐related risk for infertility (low, moderate, or high risk) was determined based on each survivor's gonadotoxic exposures, using the risk stratification consensus available at the time of patient counseling.^27^ Hormonal laboratory results were abstracted and grouped into a binary variable within or outside normal limits; values outside of normal limits for males included follicle‐stimulating hormone (FSH) ≥12.0 IU/ml^34^ and for females included either an FSH ≥40.0 IU/ml and/or an anti‐Mullerian hormone level below the lower limit of the assay‐ and age‐specific reference range.

#### 2.1.2 Fertility‐related variables

The fertility‐focused discussions were documented in the medical record using a standardized note. Interest in FSA was categorized as expressed interest (interested now or in the future [*n* = 126]) versus no expressed interest (documentation that survivor was not interested [*n* = 46] or absence of documentation regarding interest [*n* = 87]). Worry about infertility was similarly characterized as expressed worry (e.g., ‘survivor reported they were worried’, [*n* = 165]) versus no expressed worry (documentation that the survivor was not worried [*n* = 84] or absence of documentation regarding worry [*n* = 10]). The number of fertility‐focused discussions (1 vs. ≥2), and receipt of FSA (documentation of semen analysis for males or consultation with a reproductive endocrinologist for females) were also abstracted. Fertility consult (*n* = 9) and fertility preservation procedure (*n* = 21) prior to gonadotoxic treatment were combined as a single variable (yes vs. no), due to low numbers.

#### 2.1.3 Statistical analysis

Descriptive statistics were utilized to examine the sociodemographic and clinical characteristics of the sample, as well as to determine the prevalence of interest in, and completion of, FSA. Participants who expressed interest in FSA were compared to those who did not express interest using Mann–Whitney *U* tests for continuous variables and Chi‐square or Fisher's exact tests for categorical variables.

Multivariable logistic regression models were used to identify factors associated with interest in FSA, compared with no expressed interest in FSA among males and females separately. Sociodemographic and clinical characteristics (see Table 1 for list of variables) were first examined using univariable logistic regression and those with *p* < 0.1 were included in a multivariable model; for variables with >5% missing data, ‘Missing’ was included as a variable category in the model. The parsimonious multivariable model was developed using stepwise backward variable elimination, only retaining variables with *p* < 0.1 in the final model. Variables in the parsimonious model with *p* < 0.05 were considered statistically significant. All analyses were conducted using R Studio version 1.3.1093.

**TABLE 1:** Participant characteristics for the entire sample and comparisons by survivor's expressed interest in a fertility status assessment among males and females

Characteristic	Entire sample		Males (*n* = 144)				Females (*n* = 115)			
	(*N* = 259)		Expressed interest (*n* = 73)		Did not express interest (*N* = 71)		Expressed interest (*N* = 53)		Did not express interest (*N* = 62)	
Age at first fertility discussion during survivorship (years)										
Mean (SD)	19.2	(1.2)	18.9	(0.9)	19.2	(1.3)	19.3	(1.5)	19.2	(1.3)
Median (range)	18.7	(18.0, 25.3)	18.6	(18.0, 21.8)	18.7	(18.0, 24.0)	18.8	(18.0, 25.3)	18.8	(18.1, 23.7)
Age at diagnosis (years)										
Mean (SD)	9.6	(5.7)	9.9	(5.5)	9.1	(5.5)	10.2	(5.7)	9.1	(6.1)
Median (range)	10.3	(0.1, 21.9)	12.2	(0.1, 18.8)	8.4	(0.3, 19.6)	12.2	(0.3, 21.0)	8.0	(0.4, 21.9)
Time from cancer treatment completion (years)										
Mean (SD)	8.0	(5.2)	7.3	(5.1)	8.4	(5.0)	7.8	(5.7)	8.6	(5.1)
Median (range)	6.7	(1.0, 21.5)	5.6	(1.5, 19.6)	8.0	(1.1, 21.5)	5.4	(1.3, 20.6)	8.0	(1.1, 18.1)
	**N**	**(%)**	**N**	**(%)**	**N**	**(%)**	**N**	**(%)**	**N**	**(%)**
Race/ethnicity										
Non‐hispanic white	150	(57.9)	42	(51.2)	40	(48.8)	31	(45.6)	37	(54.4)
Black	67	(25.9)	18	(45.0)	22	(55.0)	15	(55.5)	12	(44.4)
Hispanic	23	(8.9)	6	(54.5)	5	(45.5)	5	(41.7)	7	(58.3)
Asian	8	(3.1)	1	(33.3)	2	(66.7)	2	(60.0)	3	(40.0)
Other	11	(4.2)	6	(75.0)	2	(25.0)	0	(0.0)	3	(100.0)
Religion										
Christian	142	(54.8)	36	(50.1)	37	(49.3)	33	(47.8)	36	(52.2)
Non‐christian or no preference	64	(24.7)	17	(45.9)	20	(54.1)	12	(44.4)	15	(55.6)
Missing	53	(20.5)	20	(58.8)	14	(41.2)	8	(42.1)	11	(57.9)
Geographical location by rural status										
Non‐rural	229	(88.4)	68	(52.3)	62	(47.7)	47	(47.5)	52	52.5)
Rural	30	(11.6)	5	(35.7)	9	(64.3)	6	(37.5)	10	(62.5)
Insurance type										
Commercial/private	180	(69.5)	50	(51.0)	48	(49.0)	43	(52.4)	39	(47.6)
Medicaid/self‐pay	79	(30.5)	23	(50.0)	23	(50.0)	10	(30.3)	23	(69.7)
Cancer diagnosis category										
Leukemia	95	(36.7)	23	(40.4)	34	(59.6)	13	(34.2)	25	(65.8)
Lymphoma	67	(25.9)	23	(63.9)	13	(36.1)	15	(48.4)	16	(51.6)
Solid tumor	97	(37.5)	27	(52.9)	24	(47.1)	25	(54.3)	21	(45.6)
Fertility preservation consult and/or procedure before treatment										
Yes	30	(11.6)	16	(66.7)	8	(33.3)	1	(16.7)	5	(83.3)
No	229	(88.4)	57	(47.5)	63	(52.5)	52	(47.8)	57	(52.3)
Hormonal laboratory evaluation^a^										
Within normal limits	199	(78.0)	55	(47.0)	62	(53.0)	34	(41.5)	48	(58.5)
Outside normal limits	56	(22.0)	17	(65.4)	9	(34.6)	16	(53.3)	14	(46.7)
Infertility risk										
Low	147	(56.8)	28	(38.9)	44	(61.1)	31	(41.3)	44	(58.7)
Moderate	41	(15.8)	15	(60.0)	10	(40.0)	8	(50.0)	8	(50.0)
High	71	(27.4)	30	(63.8)	17	(36.2)*	14	(58.3)	10	(41.7)
Number of fertility discussions during survivorship										
1	164	(63.3)	37	(39.4)	57	(60.6)	25	(35.7)	45	(64.3)
≥2	95	(36.7)	36	(72.0)	14	(28.0)***	28	(62.2)	17	(37.8)**
Survivor expressed worry about future infertility^b^										
Yes	165	(63.7)	52	(63.4)	30	(36.6)	46	(55.4)	37	(44.6)
No	94	(36.3)	41	(66.1)	21	(33.9)***	7	(21.9)	25	(78.1)**

Proportions are presented as row percentages. Differences in interest in fertility status assessment by sex were assessed using chi‐square or fisher's exact test for categorical data and *t*‐test or Mann–Whitney *U* for continuous data.

Abbreviation: SD, standard deviation.

^a^ Proportions were calculated by excluding missing values (*n* = 4). Hormonal laboratory values outside of normal limits for males included a follicle stimulating hormone (FSH) ≥12.0 and for females included either an FSH ≥40.0 and/or an anti‐Mullerian hormone level below the lower limit of the assay‐specific reference range.

^b^ Survivors were asked if they were worried about future infertility as part of the fertility discussion.

*p < 0.05; **p < 0.01; ***p < 0.001.

## 3 RESULTS

**FIGURE 1:**
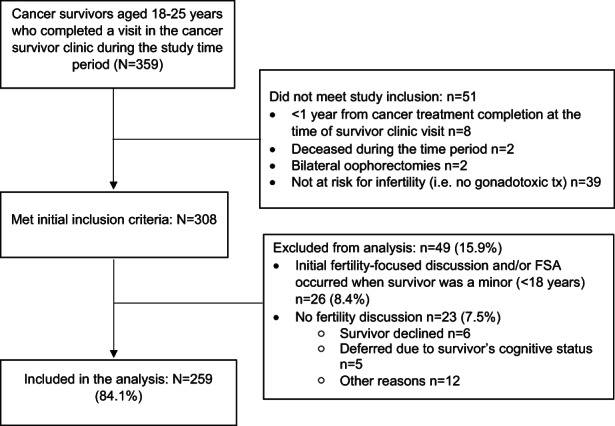
Study inclusion flow chart.

Among 308 participants who met eligibility criteria, 259 (84.1%) received a fertility‐focused discussion and were included in this analysis. Reasons for exclusion from the analysis included receiving initial fertility‐focused discussions and/or FSA when the survivor was a minor (e.g., <18 years of age; *n* = 26), no fertility discussion during survivorship visits due to patient declining the conversation (*n* = 6), other reasons (*n* = 12), or the fertility discussion was deferred due to patient's cognitive status (*n* = 5; Figure 1). There were no differences in sex or race between included and excluded participants; survivors of leukemia were underrepresented in the participant sample (36.7% of participants vs. 53.1% of non‐participants) while survivors of lymphoma were overrepresented (25.9% of participants vs. 10.2% of non‐participants, *p* = 0.028). The final sample consisted of 144 (55.6%) male and 115 (44.4%) female participants.

**FIGURE 2:**
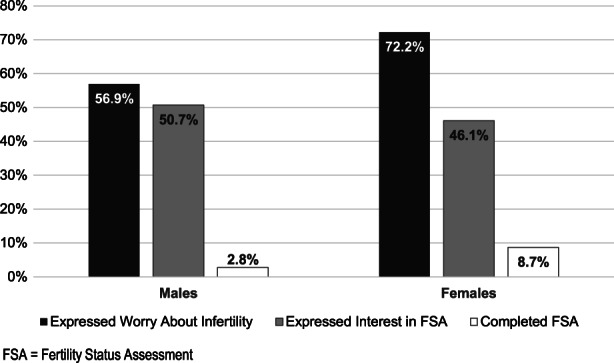
Proportion of participants who expressed worry about infertility, interest in FSA, and who completed an FSA (*N* = 259).

At the time of initial fertility discussion, participants were an average of 19.2 (±1.2) years of age and 8.0 (±5.2) years from cancer treatment completion (Table 1). The majority of participants were non‐Hispanic white (57.9%), Christian (54.8%), had commercial or private insurance (69.5%), and lived in a non‐rural area (88.4%). The most common cancer diagnosis category was a solid tumor (37.5%), followed by leukemia (36.7%) and lymphoma (25.9%). Just over half of the participants had a low level of treatment‐related infertility risk (56.8%), 15.8% had moderate risk, and 27.4% of participants were at high‐risk for infertility. The majority of participants (63.3%) completed one fertility‐focused discussion during the study period. Nearly all participants had hormonal laboratory evaluations to guide the discussion with 18.2% of males and 26.8% of females having results outside the normal limits. Worry about future infertility was expressed by 83 (72.2%) females and 82 (56.9%) males (Figure 2).

A total of 126 (48.6%) participants expressed interest in FSA with similar rates between males (*n* = 73, 50.7%) and females (*n* = 53, 46.1%). Univariable analysis of factors associated with interest in an FSA among males and females are presented in Tables S1 and S2, respectively. In a multivariable logistic regression model, factors associated with interest in FSA among males included ≥2 fertility discussions during survivorship (odds ratio [OR] 3.78, 95% confidence interval [CI]: 1.70–8.75, *p* < 0.001), fertility preservation consult and/or procedure before gonadotoxic treatment (OR = 3.02, 95%CI: 1.09–9.04, *p* = 0.039), worry about future infertility (OR = 2.40, 95%CI: 1.11–5.27, *p* = 0.026), and high level (compared with low level) of risk for infertility (OR = 2.47, 95%CI: 1.07–5.87, *p* = 0.036; Table 2). Among females, ≥2 fertility discussions during survivorship (OR = 2.45, 95%CI: 1.08–5.68, *p* = 0.033) and worry about future infertility (OR = 4.37, 95%CI: 1.71–12.43, *p* = 0.003) were related to interest in FSA (Table 3); insurance type met criteria to remain in the multivariable model but did not meet statistical significance.

**TABLE 2:** Multivariable parsimonious model of factors related to expressed interest in fertility status assessment among males (*N* = 144)

Variable	OR	95% CI		*p* value
Number of fertility discussions during survivorship				
1	1.00			
**≥2**	**3.78**	**1.70**	**8.75**	**0.001**
Fertility preservation consult and/or procedure before treatment				
No	1.00			
**Yes**	**3.02**	**1.09**	**9.04**	**0.039**
Expressed worry about infertility				
No	1.00			
**Yes**	**2.40**	**1.11**	**5.27**	**0.026**
Infertility risk				
Low	1.00			
Moderate	1.86	0.67	5.30	0.236
**High**	**2.47**	**1.07**	**5.87**	**0.036**

Using backwards elimination, cancer diagnosis was eliminated from the parsimonious model.

**TABLE 3:** Multivariable parsimonious model of factors related to expressed interest in fertility status assessment among females (*N* = 115)

Variable	OR	95% CI		*p* value
Number of fertility discussions during survivorship				
1	1.00			
**≥2**	**2.45**	**1.08**	**5.68**	**0.033**
Expressed worry about infertility				
No	1.00			
**Yes**	**4.37**	**1.71**	**12.43**	**0.003**
Insurance type				
Commercial/private	1.00			
Medicaid/self‐pay	0.40	0.16	0.99	0.053

Using backwards elimination, cancer diagnosis, and hormonal laboratory evaluation were eliminated from the parsimonious model.

Only 4 (2.8%) males and 10 (8.7%) females survivors had completed FSA while being followed in the cancer survivor clinic. Models for FSA completion could not be tested/built due to the relatively low rates of completion.

## 4 DISCUSSION

For survivors of childhood cancer who are at risk for infertility, FSA can provide clarity regarding their current fertility status and options for future biological parenthood. In this study of YASCC, who had received a fertility‐focused discussion during the study window, about half were interested in FSA (51% of males, 46% of females), although few had completed assessments (3% of males, 9% of females) before transitioning from our institution to adult healthcare. Prior research has focused on a wide age range of YASCC while our study adds to the literature by demonstrating a large proportion of survivors are interested in FSA during emerging adulthood. Additionally, we utilized an approach that increases the ecological validity of the study findings, as data were abstracted from standardized notes in the medical record and reflected clinical encounters that occurred in the survivorship clinic. Among both males and females, worry about infertility and more than one fertility‐focused discussion during survivorship were associated with interest in FSA; for males, additional factors associated with interest in FSA included being at high‐risk for infertility and having completed a fertility consultation and/or fertility preservation procedure prior to their gonadotoxic treatment.

Prior studies of YASCC consistently demonstrate that survivors are interested in information regarding their risk for infertility and education about reproductive health.^24^, ^27^ We found that, despite half of the sample reporting interest, less than 10% of survivors had received an FSA. Our findings are consistent with previous research which suggests that while survivors are interested in FSA, few have pursued these services.^26^, ^29^, ^30^ YASCC who have pursued FSA described a desire to be prepared for the future, while others have not pursued assessment because they are worried about the finality of the results if they are infertile.^35^ The lack of FSA completion in our study may be influenced by the young age of participants in our sample, many of whom are likely years from pursuit of a pregnancy or family‐building. Additionally, over half (58%) of survivors received gonadotoxic treatment that is associated with low‐risk for infertility, and three‐quarters (78%) had normal hormonal evaluations, which may be reassuring to survivors, resulting in a delay of pursuit for FSA.Male and female survivors who were worried about future infertility had a 3‐ and 4‐fold increased likelihood of expressing interest in FSA, respectively. Reproductive concerns, including worry, are common among cancer survivors and can be associated with uncertain fertility status. FSA may provide insight regarding future reproductive health choices and options for biological parenthood for survivors who are interested. Uncertain fertility status is reported by 48–77% of survivors of childhood cancer, despite an overwhelming interest in biological children.^26^, ^29^, ^31^, ^32^ In qualitative studies of young adult survivors, females describe the psychological burden of uncertain fertility, pressure on their family building timeline due to possible premature menopause, and missing out on the shared peer and social experiences associated with pregnancy.^36^, ^37^ In our study, survivors were provided with information to access FSA, however, few have completed an assessment. The decision‐making process for FSA and barriers and facilitators to accessing FSA among emerging adult survivors warrants further study.

FSA procedures and results differ for males and females. Males who are at least several years from cancer treatment completion and complete a semen analysis can generally receive a confirmation of fertility or infertility which is unlikely to change as they age. The costs for this procedure are generally <USD $300. For females, results of FSA can provide insight into their current fertility status and reproductive window, but findings are less definitive and females' fertility status changes with age. There is also the potential for higher costs associated with hormonal laboratory evaluation, transvaginal ultrasound to measure antral follicle count, and consultation with the reproductive endocrinologist if insurance coverage is not available. There may be potential adverse psychological consequences for survivors who pursue FSA and receive results that suggest infertility or likelihood of reduced fertility. For example, survivors may experience a sense of loss, regret for not taking part in fertility preservation at the time of cancer diagnosis, or worry regarding perceived social consequences of infertility.^7^ However, receiving an FSA earlier, during emerging adulthood, may be helpful by providing more time for survivors to pursue alternative parenthood options and reduce distress associated with uncertainty.^36^ For females, FSA during emerging adulthood can be particularly informative for survivors who will likely have a shortened reproductive window by offering an opportunity for post‐treatment fertility preservation. However, these assessments may be burdensome to some survivors, with the potential for a negative impact on romantic relationships and could lead some to feel pressured to have children before they are ready.^32^, ^35^

For both males and females, two or more fertility‐related discussions were associated with interest in FSA. For some survivors, the information provided in the initial discussion may be overwhelming to process, meaning the survivor may not become interested in FSA until a second or third fertility discussion. For other survivors who are interested in FSA, repeated fertility discussions during survivor clinic visits may be relevant. In our survivor clinic, fertility discussions are generally initially offered at age 18 years, offered again at the time of transition to adult healthcare (age 21 years), and offered/available at any other time if the patient has questions. Cancer survivors have previously expressed strong desires for fertility‐focused education and discussions after cancer treatment,^24^, ^29^ and offering these discussions more than once has been helpful to survivors who are interested. Most pediatric cancer centers transition survivors to adult healthcare at some point during emerging adulthood, which can coincide with the time when survivors are most interested in pursuing FSA. Having repeated discussions prior to transition may help survivors be aware of how to access reproductive health services when desired.

Among males, being at high‐risk for treatment‐related infertility and the receipt of a fertility preservation consult and/or fertility preservation procedure at the time of cancer diagnosis were associated with interest in FSA. Those at high risk for infertility may be aware of this risk and more motivated to determine if their cancer treatment did indeed result in infertility. Treatment‐related infertility risk was not associated with interest in FSA among females. Prior research demonstrates that females can have perceptions of infertility risk that are discordant from their treatment exposures and/or counseling.^20^ It is possible that females may be more motivated to pursue an FSA based on their desire for future pregnancy or parenthood, rather than their treatment‐related risk factors. Further research is needed to explore decisional factors related to female survivors' pursuit of an FSA. Prior receipt of fertility consult, or participation in sperm banking, may set the stage for survivors to be familiar with reproductive health services and the semen analysis process. Previous research among adult men who banked sperm at the time of cancer diagnosis has found that perceived intensity of treatment and experiences with sperm banking were related to semen analysis monitoring after cancer treatment.^28^ Although not statistically significant, having public insurance or no insurance was associated with lower odds of interest in FSA among females. Healthcare structural barriers, including the role of insurance coverage, perceived costs, and knowledge and comfort in navigating reproductive health clinics are important areas for future study.

### 4.1 Limitations

While this study presents important findings related to interest in FSA among YASCC, there are limitations that need to be considered. The data from this study were obtained from a single site, through chart review of healthcare provider documentation of fertility discussions. It is possible that some survivors received an FSA that was not documented in our medical records. Similarly, survivors' worry about future infertility and interest in FSA may differ if assessed via self‐report, compared with the provider's documentation of their discussion. As these data stem from clinical discussions, we recognize that there may be heterogeneity in how rigorously these outcomes were assessed and documented, with potential variations by the practitioner, which may have led to reporting bias. Worry was dichotomized for this analysis, which does not reflect the range of worry among YASCC. Most survivors in our sample had hormonal laboratory evaluations at some point after cancer treatment and it is possible that those results affected the FSA completion rates. The sample included an underrepresentation of survivors of leukemia and an overrepresentation of survivors of lymphoma, which may have impacted the findings regarding interest in FSA. Survivors of brain tumors were excluded from this study. We excluded survivors who did not receive gonadotoxic treatment, however, prior research has demonstrated that survivors may worry about their fertility status irrespective of their treatment exposures.^20^ Prior research at our institution found 70% of eligible patients successfully transition from active treatment clinic to our survivor clinic^38^ and nearly 90% of our established survivors return to our survivorship clinic for annual care,^39^ however, some survivors may not attend survivor clinic, or may be lost to follow‐up, and therefore not receive a fertility‐focused discussion at 18 years of age. Finally, we excluded survivors who initiated a fertility discussion/FSA while they were minor, which involved a more direct level of parental input and consent than those who began these discussions during emerging adulthood. The strengths of this study include a diverse sample of survivors from racial and ethnic minority groups and those with public insurance.

Future research in this area is warranted to explore the predictive validity of interest in FSA in relation to FSA completion. As reproductive health has become a focus of cancer survivorship care, there is a need to develop interventions to assist survivors in accessing reproductive health services to optimize options for parenthood. Within the context of emerging adulthood, survivors are experiencing changes and transitions in healthcare, school, work, and family life. This is a time period with the potential for high reproductive health needs, and developmentally appropriate interventions are needed to support survivors' decisions to pursue FSA.

In conclusion, this study highlights an important discrepancy in reproductive healthcare among YASCC. Most survivors are worried about future infertility and half report interest in FSA, yet few have received an FSA. Future research is needed to explore barriers to accessing reproductive health services and decision‐making related to FSA. Counseling survivors regarding their risk for infertility and providing referrals for FSA may benefit survivors in emerging adulthood.

## AUTHOR CONTRIBUTIONS

**Brooke Cherven**: conceptualization, data curation, formal analysis, funding acquisition, investigation, methodology, project administration, resources, software, supervision, validation, visualization, writing ‐ original draft, and writing ‐ review and editing.

**Rebecca Williamson Lewis**: conceptualization, data curation, formal analysis,, methodology, project administration, software, validation, visualization, writing ‐ original draft, and writing ‐ review and editing.

**Megan Pruett**: conceptualization, data curation, writing ‐ original draft, and writing ‐ review and editing.

**Lillian Meacham**: conceptualization, data curation, funding acquisition, investigation, methodology, validation, visualization, writing ‐ original draft, and writing ‐ review and editing.

**James Klosky**: conceptualization, data curation, formal analysis, funding acquisition, investigation, methodology, resources, validation, visualization, writing ‐ original draft, and writing ‐ review and editing.

## FUNDING INFORMATION

This Investigator‐Initiated Nursing Research Project was supported by the Children's Oncology Group National Clinical Trials Network Operations Grant (U10CA180886; PI: Hawkins). Research reported in this publication was also supported by the National Institute Of Nursing Research of the National Institutes of Health under Award Number K23NR020037. The content is solely the responsibility of the authors and does not necessarily represent the official views of the National Institutes of Health.

## CONFLICT OF INTEREST

The authors report no conflicts of interest.

## ETHICAL STATEMENT

This study received ethical approval from the Children's Healthcare of Atlanta institutional review board (IRB# 00000416), and was conducted in accordance with the Declaration of Helsinki.

## Supporting information


Table S1‐S2

## Data Availability

The data that support the findings of this study are available from the corresponding author upon reasonable request.
