# Supplementary material for: Interest in fertility status assessment among young adult survivors of childhood cancer
Source: Cancer Med. 2022 Jun 1;12(1):674–83. doi: 10.1002/cam4.4887 (PMC9844611; doi:10.1002/cam4.4887)
Supplement: Supplementary file 1 — Table S1‐S2 [file CAM4-12-674-s001.docx]

Supplemental Table 1. Univariable analysis of factors associated with interest in a fertility status assessment among male participants (N=144)

| Variable | OR | 95% CI | | P value |
| --- | --- | --- | --- | --- |
| Age at first survivorship fertility discussion (years) |  |  |  |  |
| 1-year increase | 0.81 | 0.58 | 1.10 | .185 |
| Age at cancer diagnosis (years) |  |  |  |  |
| 1-year increase | 1.03 | 0.97 | 1.10 | .387 |
| Time from cancer treatment completion (years) |  |  |  |  |
| 1-year increase | 0.95 | 0.89 | 1.02 | .169 |
| Race |  |  |  |  |
| NH White | 1.00 |  |  |  |
| NH Black | 0.78 | 0.36 | 1.66 | .519 |
| Hispanic | 1.14 | 0.32 | 4.25 | .836 |
| Asian | 0.48 | 0.02 | 5.16 | .551 |
| Other | 2.86 | 0.62 | 20.29 | .215 |
| Religion |  |  |  |  |
| Christian | 1.00 |  |  |  |
| Non-Christian or No Preference | 0.87 | 0.39 | 1.93 | .738 |
| Missing | 1.47 | 0.65 | 3.39 | .360 |
| Geographical location by rural status |  |  |  |  |
| Non-rural | 1.00 |  |  |  |
| Rural | 0.51 | 0.15 | 1.55 | .245 |
| Insurance type |  |  |  |  |
| Commercial/Private | 1.00 |  |  |  |
| Medicaid/Self-Pay | 0.96 | 0.48 | 1.94 | .909 |
| Cancer Diagnosis |  |  |  |  |
| Leukemia | 1.00 |  |  |  |
| Lymphoma | 2.62 | 1.12 | 6.32 | .029 |
| Solid Tumor | 1.66 | 0.78 | 3.60 | .191 |
| Fertility preservation attempt/consult pre-treatment |  |  |  |  |
| No | 1.00 |  |  |  |
| Yes | 2.21 | 0.90 | 5.81 | .092 |
| Hormonal laboratory evaluation^†^ |  |  |  |  |
| Within normal limits | 1.00 |  |  |  |
| Outside normal limits | 2.13 | 0.90 | 5.36 | .095 |
| Infertility Risk Level |  |  |  |  |
| Low | 1.00 |  |  |  |
| Moderate | 2.36 | 0.94 | 6.13 | .071 |
| High | 2.77 | 1.31 | 6.03 | .008 |
| Number of survivorship fertility discussions |  | | | |
| 1 | 1.00 |  |  |  |
| ≥2 | 3.96 | 1.92 | 8.54 | <.001 |
| Expressed worry about infertility |  |  |  |  |
| No/not documented | 1.00 |  |  |  |
| Yes | 3.38 | 1.71 | 6.86 | <.001 |

Note. Variables that were significant at p<.10 were tested in the multivariable model.

^†^Hormonal laboratory values outside of normal limits for males included a follicle stimulating hormone (FSH) ≥12.0 and for females included either an FSH ≥40.0 and/or an anti-Mullerian hormone level below the lower limit of the assay-specific reference range.

Supplemental Table 2. Univariable analysis of factors associated with interest in a fertility status assessment among female participants (N=115)

| Variable | OR | 95% CI | | P value |
| --- | --- | --- | --- | --- |
| Age at first survivorship fertility discussion (years) |  |  |  |  |
| 1-year increase | 1.05 | 0.80 | 1.39 | .697 |
| Age at cancer diagnosis (years) |  |  |  |  |
| 1-year increase | 1.03 | 0.97 | 1.20 | .328 |
| Time from cancer treatment completion (years) |  |  |  |  |
| 1-year increase | 0.97 | 0.90 | 1.04 | .399 |
| Race |  |  |  |  |
| NH White | 1.00 |  |  |  |
| NH Black | 1.49 | 0.61 | 3.71 | .382 |
| Hispanic | 0.85 | 0.23 | 2.94 | .801 |
| Asian | 0.80 | 0.10 | 5.09 | .809 |
| Other^±^ | -- | -- | -- | -- |
| Religion |  |  |  |  |
| Christian | 1.00 |  |  |  |
| Non-Christian or No Preference | 0.87 | 0.35 | 2.13 | .765 |
| Missing | 0.79 | 0.28 | 2.20 | .658 |
| Geographical location by rural status |  |  |  |  |
| Non-rural | 1.00 |  |  |  |
| Rural | 0.66 | 0.21 | 1.93 | .460 |
| Insurance type |  |  |  |  |
| Commercial/Private | 1.00 |  |  |  |
| Medicaid/Self-Pay | 0.39 | 0.16 | 0.91 | .034 |
| Cancer Diagnosis |  |  |  |  |
| Leukemia | 1.00 |  |  |  |
| Lymphoma | 1.80 | 0.69 | 4.84 | .235 |
| Solid Tumor | 2.29 | 0.95 | 5.67 | .067 |
| Fertility preservation attempt/consult pre-treatment |  |  |  |  |
| No | 1.00 |  |  |  |
| Yes | 0.22 | 0.01 | 1.42 | .172 |
| Hormonal laboratory evaluation^†^ |  |  |  |  |
| Within normal limits | 1.00 |  |  |  |
| Outside normal limits | 1.61 | 0.70 | 3.78 | .265 |
| Infertility Risk Level |  |  |  |  |
| Low | 1.00 |  |  |  |
| Moderate | 1.42 | 0.47 | 4.26 | .526 |
| High | 1.99 | 0.79 | 5.17 | .149 |
| Number of survivorship fertility discussions |  | | | |
| 1 | 1.00 |  |  |  |
| ≥2 | 2.96 | 1.38 | 6.55 | .006 |
| Expressed worry about infertility |  |  |  |  |
| No/not documented | 1.00 |  |  |  |
| Yes | 4.44 | 1.80 | 12.17 | .002 |

Note. Variables that were significant at p<.10 were tested in the multivariable model.

^±^Results for the Other race category were uninterpretable due to low numbers (0/3 expressed interest in a fertility status assessment).

^†^Hormonal laboratory values outside of normal limits for males included a follicle stimulating hormone (FSH) ≥12.0 and for females included either an FSH ≥40.0 and/or an anti-Mullerian hormone level below the lower limit of the assay-specific reference range.
